# A Compact MIMO Multiband Antenna for 5G/WLAN/WIFI-6 Devices

**DOI:** 10.3390/mi14061153

**Published:** 2023-05-30

**Authors:** Ayyaz Ali, Mehr E Munir, Mohamed Marey, Hala Mostafa, Zahriladha Zakaria, Ahmed Jamal Abdullah Al-Gburi, Farooq Ahmed Bhatti

**Affiliations:** 1 Department of Electrical Engineering, Military College of Signals, National University of Sciences and Technology, Islamabad 44000, Pakistan; 2 Smart Systems Engineering Laboratory, College of Engineering, Prince Sultan University, Riyadh 11586, Saudi Arabia; 3 Electrical Engineering Department, Iqra National University, Peshawar 25000, Pakistan; 4 Department of Information Technology, College of Computer and Information Sciences, Princess Nourah bint Abdulrahman University, P.O. Box 84428, Riyadh 11671, Saudi Arabia; 5 Centre of Telecommunication Research & Innovation (CeTRI), Fakulti Kejuruteraan Elektronik dan Kejuruteraan Komputer (FKEKK), Universiti Teknikal Malaysia Melaka, Melaka 76100, Malaysia; 6 Center for Telecommunication Research & Innovation (CeTRI), Faculty of Electrical and Electronic Engineering Technology (FTKEE), Melaka 76100, Malaysia

**Keywords:** MIMO antenna, multiband, 5G, WIFI-6

## Abstract

This research work presents a compact design of a Multiple-Input Multiple-Output (MIMO) multiband antenna along with high-isolation characteristics. The presented antenna was designed for 3.50 GHz, 5.50 GHz, and 6.50 GHz frequencies for 5G cellular, 5G WiFi, and WiFi-6, respectively. The fabrication of the aforementioned design was undertaken using FR-4 (1.6 mm thickness) substrate material with a loss tangent and relative permittivity of about 0.025 and 4.30, respectively. The two-element MIMO multiband antenna was miniaturized to 16 × 28 × 1.6 mm^3^, making it desirable for devices operating in 5G bands. High isolation (>15 dB) was attained with thorough testing without employing a decoupling scheme in the design. Laboratory measurements resulted in a peak gain of 3.49 dBi and an efficiency of around 80% in the entire operating band. The evaluation of the presented MIMO multiband antenna was carried out in terms of the envelope correlation coefficient (ECC), diversity gain (DG), total active reflection coefficient (TARC), and Channel Capacity Loss (CCL). The measured ECC was less than 0.04, and the DG was well above 9.50. The observed TARC was also lower than −10 dB, and the CCL was below 0.4 bits/s/Hz in the entire operating band. The presented MIMO multiband antenna was analyzed and simulated using CST Studio Suite 2020.

## 1. Introduction

In today’s wireless communication technology and 5G era, in which wireless devices are evolving at a very fast pace, the improvement of data transmission with large capacity and link reliability is greatly needed [1]. Researchers have proposed various methods to meet demands, and MIMO technology has turned out to be one of the most fruitful solutions for enhancing the data rate [2]. However, to achieve high isolation, high gain, and compact size, one needs to overcome many design challenges. Over the past few years, researchers have presented numerous MIMO antennas in the literature. A tri-band coplanar MIMO antenna was presented in [3] for WiMAX and WLAN applications. Its single-element design consisted of a monopole antenna with two L-shaped arms and a meandered microstrip line. It had a compact size of 48 × 48 × 1.6 mm^3^ and was fabricated on an FR-4 substrate, but it offered low gain and isolation values. In MIMO antennas, it is critical to maintain high isolation between the radiating elements for good radiation performance of the MIMO system. A simple approach to achieve isolation of less than −10 dB using ferrite skin was presented in [4]. Two inverted F-shaped antenna elements radiating at 3.5 GHz were placed 1.4 mm apart. The ferrite coating was applied to the radiating element to improve the isolation between the antenna elements. An increase in isolation of 3.79 dB was reported by carefully controlling the ferrite material coating and its thickness. The impedance bandwidth was also improved to 0.14 GHz. This paper presented a simple technique for improving isolation in a MIMO antenna, but the method did not provide enough isolation and reduced the gain and radiation efficiency of the antenna. A four-port wideband MIMO antenna was designed to operate in the frequency range of 4.36–6.90 GHz [5]. It had a simple monopole on the front side and a ground plane with circular slits etched on the back side of the substrate. High isolation was achieved by placing the antenna elements in an orthogonal position to each other. It had a peak gain of 2 dB and a radiation efficiency higher than 90%. This MIMO antenna had good performance in terms of impedance bandwidth, isolation, and radiation efficiency, but it had low gain and a high ECC value. A dual-port, dual-polarized MIMO antenna for 5G and WLAN communication systems was presented in [6]. It was fabricated on an FR-4 substrate with a stepped microstrip line on the top side and a square ground structure on the bottom side. The isolation was enhanced by designing a passive decoupling structure between the antenna elements and etching three slots on the ground plane. It operated in the frequency ranges of 3.4–3.6 GHz and 5.15–5.85 GHz. Although this MIMO design had a compact antenna and provided dual-band operation, it had low gain and isolation and a high ECC value. A compact MIMO antenna resonating at 3.1 to 5.2 GHz frequency bands was presented in [7] for WiMAX and WLAN systems. It consisted of two different antenna designs: Antenna 1 was a modified T-shaped strip line, while Antenna 2 was a square-shaped microstrip electromagnetically coupled to its feedline. The ground plane was a slotted square strip. The orthogonal orientation of the antenna elements provided isolation between them. While the presented antenna had a wide frequency coverage, it offered low gain and isolation. Another technique to improve isolation is to modify the ground structure, which was presented in [8], in which L-shaped and T-bar-shaped structures were used. Moreover, the orthogonal mode method and an isolator designed with a circular structure providing high isolation were presented in [9]. An AI-based MIMO antenna design was presented in terms of return loss, radiation patterns, and diversity gain. The Genetic Algorithm (GA) and Neural Network (NN) were used to improve antenna performance; in addition, the effect of mutual coupling between antenna elements was investigated using AI techniques [10]. A deep learning-based MIMO antenna selection and Channel State Information (CSI) extrapolation approach was used to investigate random selection and linear interpolation in terms of spectral efficiency and complexity [11]. An overview of artificial intelligence (AI) approaches in antennas for environmental sensing was presented. The authors briefly explained AI techniques in antenna design through several case studies in which AI-based antennas were used for environmental sensing applications [12]. A novel approach using artificial neural networks (ANNs) to design a microstrip patch antenna for radar applications was presented. The proposed approach involved training an ANN to predict the optimal dimensions of the microstrip patch antenna based on the desired operating frequency and other performance parameters; in addition, a good comparison was provided between the performance of the proposed approach and that of traditional design methods, such as the Transmission Line Model (TLM) and the Finite Element Method (FEM) [13]. In this research, different machine learning techniques utilized by several case studies on antenna design and optimization, such as artificial neural networks, support vector machines, and genetic algorithms, were presented [14]. A detailed investigation of recent developments and technical solutions in the design and development of innovative space–air–ground integrated networks to support seamless and ubiquitous wireless connectivity for future 6G wireless communications was presented. The authors addressed different application scenarios and implementation challenges for SAGI 6G networks [15]. The performance analysis of Multiple-Input Multiple-Output (MIMO) systems using circular polarization was presented. The authors investigated how circular polarization affects MIMO system metrics, such as capacity, diversity gain, and channel correlation. In addition, the research suggested a novel strategy for optimizing the design of circularly polarized antennas for MIMO systems. The suggested method calculated the optimal antenna rotation angles using the channel matrix’s Singular Value Decomposition (SVD) [16]. A novel design of mm-wave circularly polarized MIMO antenna array was presented that performed effectively for indoor 5G applications at 28 GHz. The proposed antenna array had good radiation patterns and diversity gain. The author used a simple decoupling network to minimize the mutual coupling between two elements [17]. A wideband 5G millimeter-wave, circularly polarized, single-layer MIMO antenna was presented. The operating bandwidth of the antenna was 25 GHz–29.5 GHz. The antenna’s radiation mechanism was thoroughly investigated using reflection phase characteristics and the transmission-line model. The MIMO antenna had high isolation between the antenna elements (<−30 dB) with a low ECC and a channel capacity loss of 0.19 bits/s/Hz [18].

The research efforts in this paper demonstrate a compact multiband 2 × 1 MIMO antenna that operates at 3.50 GHz, 5.50 GHz, and 6.50 GHz, catering to 5G, WLAN, and WiFi-6 devices, respectively. Initially, a single antenna element was introduced, and subsequently, the design was replicated orthogonally for a 2 × 1 MIMO configuration. The notable features of this antenna include its higher gain, enhanced radiation efficiency, superior isolation, improved diversity performance, and reduced antenna size in comparison with the antennae in the existing literature. The primary objective behind the design of the proposed MIMO antenna was to offer a compact design with simplified fabrication, suitable for devices operating within the 5G frequency bands that require compact antennas.

## 2. Single Antenna Element Design

**Figure 1:**
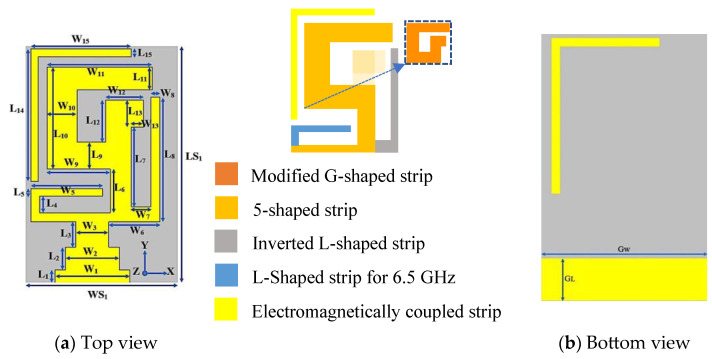
Top and bottom illustration of the single antenna element.

**Figure 2:**
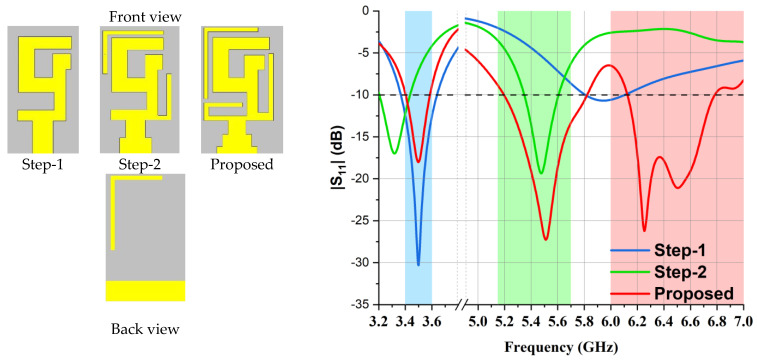
Simulated S11 results and design steps of single-element antenna.

In this section, a single element of the MIMO multiband antenna design is explained. Its geometry is presented in Figure 1. The measurements of the single antenna element were 16 × 10 × 1.6 mm^3^, and it was fabricated on an FR4 (1.6 mm thickness) substrate (ε_r_ = 4.30 and tan(δ) = 0.025). It was fed through microstrip lines of distinct lengths and widths to achieve good multiband characteristics. On the back side of the substrate, there was a partial ground structure and a flipped L-shaped strip, which provided a wide impedance bandwidth for the 6.5 GHz frequency. Its simulated reflection coefficient (S_11_) along with the design steps are presented in Figure 2.

Firstly, a simple 5G-shaped strip with a modified G-shape (embedded within it) along with a microstrip feeding line was designed on the uppermost side, and a partial or semi-ground structure was etched on the lowermost side of the substrate. The 5G-shaped strip was tuned to radiate at 3.5 GHz. At its optimized version, the reflection coefficient for the 3.5 GHz band was well below −10 dB. In step 2, an inverted L-shaped strip was added to acquire the categorization of a dual-band, i.e., 3.50 GHz and 5.50 GHz. The feeding structure was modified for a better impedance bandwidth (<−10 dB); these modifications shifted the 3.5 GHz dip, which was rectified in step 3. For the proposed antenna design, two L-shaped strips were added, one at the bottom side of the substrate and the other at the bottom edge of the 5G-shaped strip. The microstrip feeding structure was tailored and resulted in triple band characteristics (3.50 GHz, 5.50 GHz, and 6.50 GHz).

**Figure 3:**
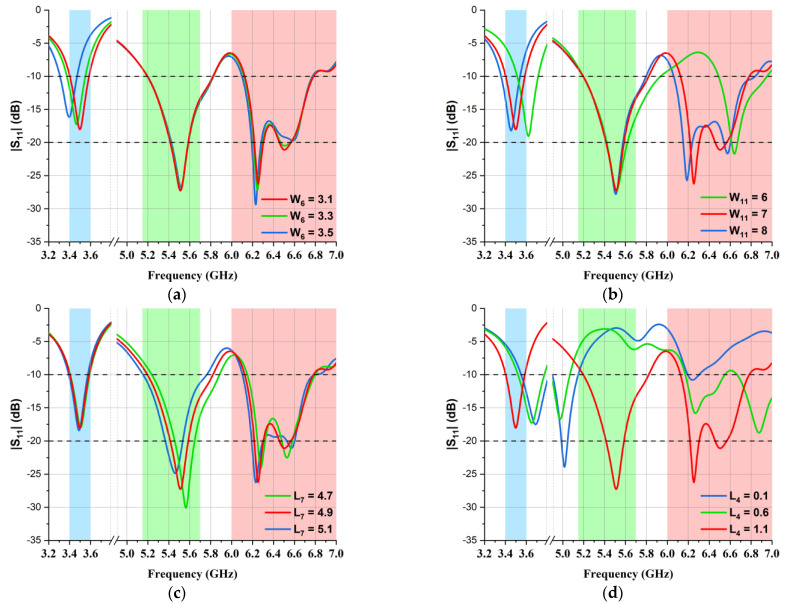

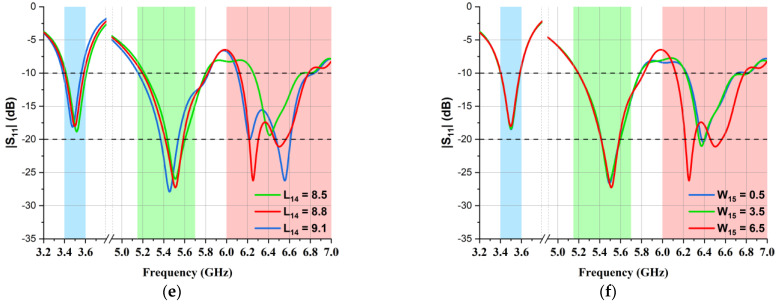
Parametric analysis of the presented MIMO multiband antenna. (**a**) W_8_; (**b**) W_11_; (**c**) L_7_; (**d**) L_4_; (**e**) L_14_; (**f**) W_15_.

**Figure 4:**
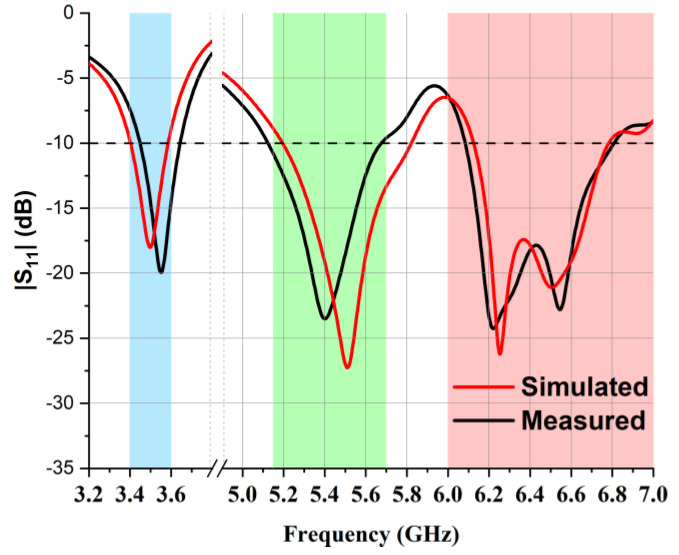
Laboratory-measured and simulated S11 of the single-element antenna.

The physical dimensions of the presented MIMO multiband antenna design are tabulated below in Table 1. Moreover, to elaborate the operation mechanism of the antenna, the parametric analysis is shown in Figure 3. As mentioned before, the 3.5 GHz band was achieved by designing the 5G-shaped strip, so any deviation from the optimized values W_6_ = 3.1 mm and W_11_ = 7 mm resulted in variations, as presented in Figure 3a and Figure 3b, respectively. In the 5.5 GHz frequency band, L_7_ was evaluated at different values, as shown in Figure 3c, and the best results were observed at L_7_ = 4.9 mm. The L-shaped strip, which was embedded to obtain the 6.5 GHz frequency band, made the other design parameters more sensitive to any change, as can be seen in Figure 3d, so after the optimization of L_4_, its finest value was observed to be L_4_ = 1.1 mm. Moreover, L_14_ and W_15_ were fine-tuned at 8.8 mm and 6.5 mm, respectively, to obtain a wide impedance bandwidth at the same frequency, as presented in Figure 3e,f, respectively. A comparison of the reflection coefficients (S_11_) of simulated and laboratory-measured single-element antennas is illustrated in Figure 4. It is evident in the graph that an impedance bandwidth below −10 dB was achieved for 3.50 GHz, 5.50 GHz, and 6.50 GHz frequency bands.

## 3. Design Analysis of MIMO Antenna

**Figure 5:**
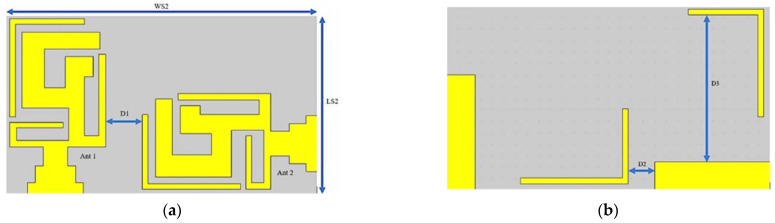
Two-element MIMO multiband antenna. (**a**) Top view and (**b**) bottom view.

To design a two-element MIMO multiband antenna, a single-element antenna was duplicated and marked as Ant 1 and Ant 2, as depicted in Figure 5. During the design process, these two antenna elements were shifted to different locations on the substrate to obtain an isolation of less than −15 dB between the antennas, which was of prime concern.

**Figure 6:**
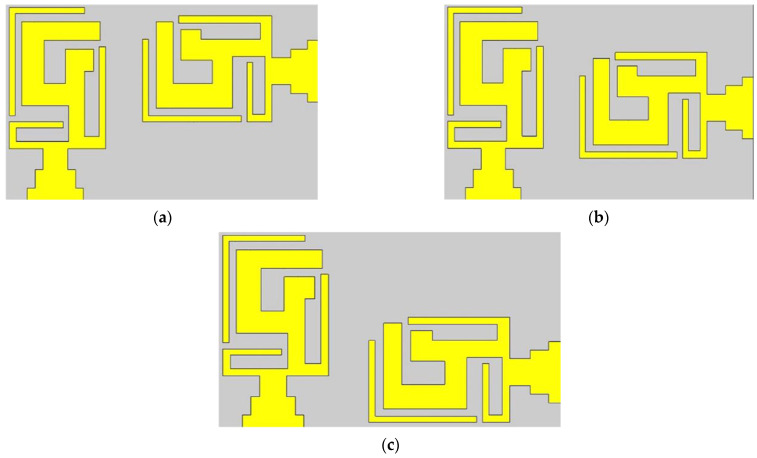
Different configurations of the two-element MIMO multiband antenna. (**a**) Top arrangement, (**b**) center arrangement, and (**c**) bottom arrangement.

**Figure 7:**
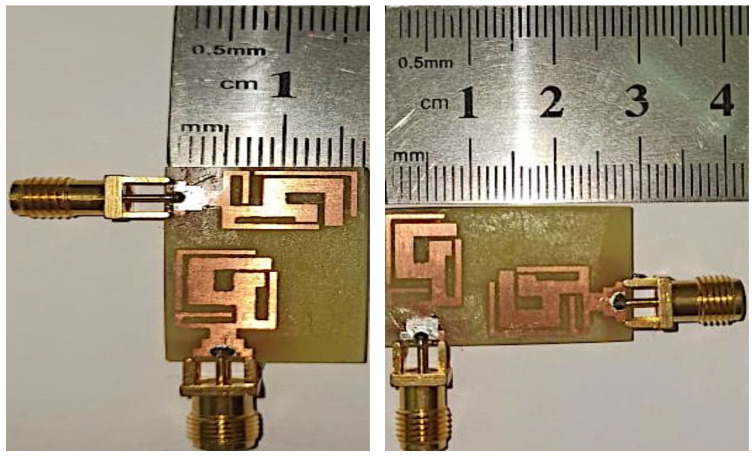
Fabricated design of two-element MIMO antenna.

**Figure 8:**
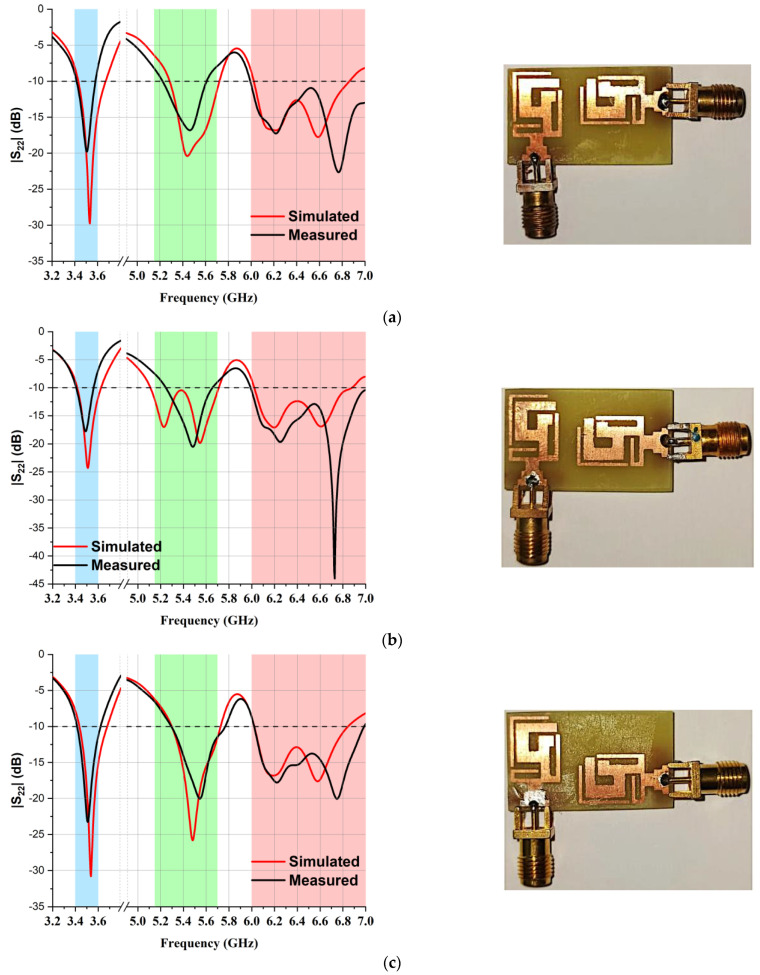
Comparison of reflection co-efficient (S22 dB) at three different positions: (**a**) top position, (**b**) center position, and (**c**) bottom position.

In the MIMO design, the minimum distance between the centers of two antennas should be λ/2 in order to obtain good isolation, but this distance would make the physical area of the MIMO design too large for a compact antenna design. The best possible alternative for this problem is to place the second element in a different orientation. An optimal distance D1 of 3.3 mm was set, which gave us the total dimensions of our proposed MIMO antenna, which were 16 × 28 mm^2^. Furthermore, Ant 2 was shifted into three different locations—the top (Figure 6a), center (Figure 6b), and bottom (Figure 6c) positions—to study the S_22_ parameter, as depicted in Figure 6. A fabricated antenna is displayed in Figure 7. Figure 8 highlights that the optimum reflection coefficient (S_22_) was observed when Ant 2 was placed in the bottom position.

**Figure 9:**
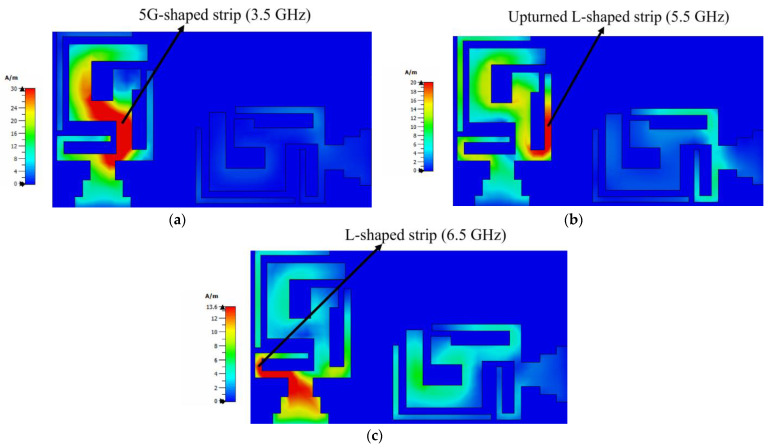
Current density of two-element MIMO multiband antenna: (**a**) 3.50 GHz, (**b**) 5.50 GHz, and (**c**) 6.50 GHz.

The simulated maximum surface current density at the resonance frequencies of a two-element multiband MIMO antenna at port 1 is depicted in Figure 9. As antenna 1 was excited, the surface current density for 3.50 GHz, 5.50 GHz, and 6.50 GHz reached the maximum around antenna 1, as shown in Figure 9a–c. It is evident in the figure that for frequencies of 3.50 GHz, the maximum current density appeared on the 5G-shaped strip; for frequencies of 5.50 GHz, the upturned L-shaped strip was resonating; and for frequencies of 6.5 GHz, the L-shaped strip embedded at the bottom edge was resonating. A small current coupling of antenna 1 with antenna 2 can be observed at 5.50 GHz and 6.50 GHz owing to higher-order mode excitation.

**Figure 10:**
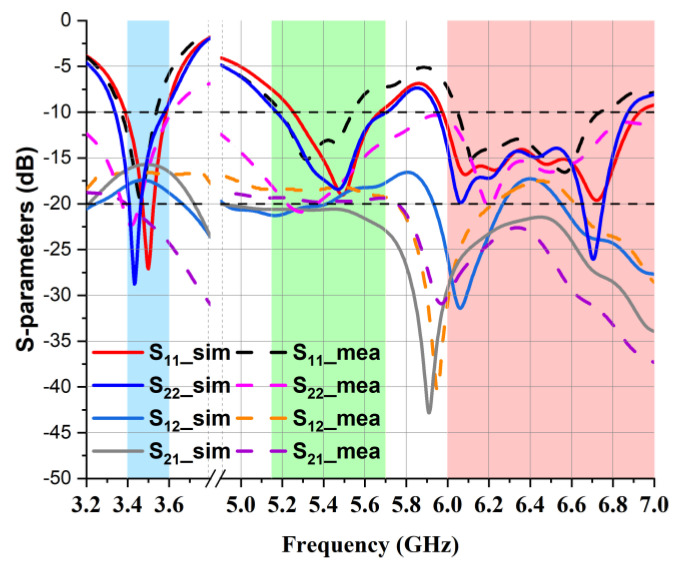
S-parameters (S11 and S12) of the presented two-element MIMO antenna.

The laboratory-measured and simulated isolation coefficient S_12_ and reflection coefficient S_11_ of the presented MIMO multiband antenna are shown in Figure 10. In the graph, it is notable that both the simulated and laboratory-measured isolation (S_12_) were below −15 dB, and the presented antenna showed good isolation in the entire operating band. This was achieved because of the inherent property of polarization diversity.

## 4. Results and Discussions

**Figure 11:**
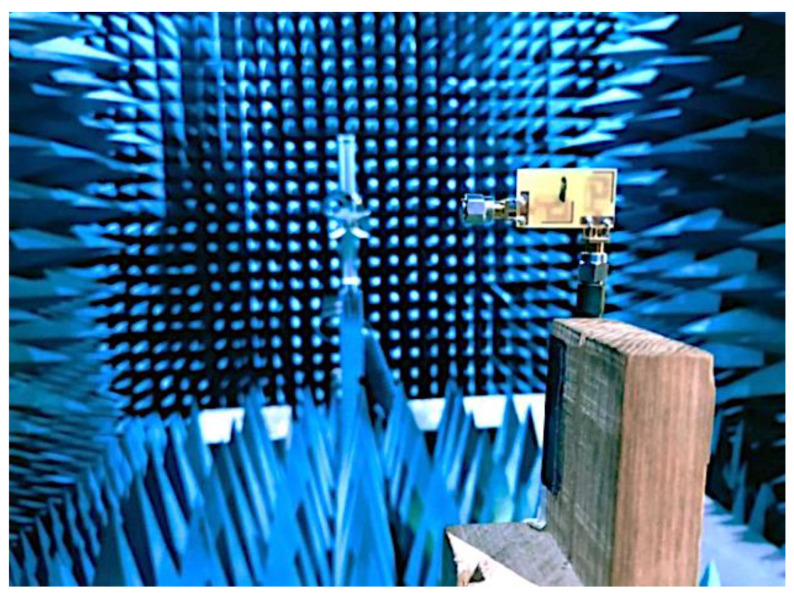
MIMO antenna anechoic chamber setup.

**Figure 12:**
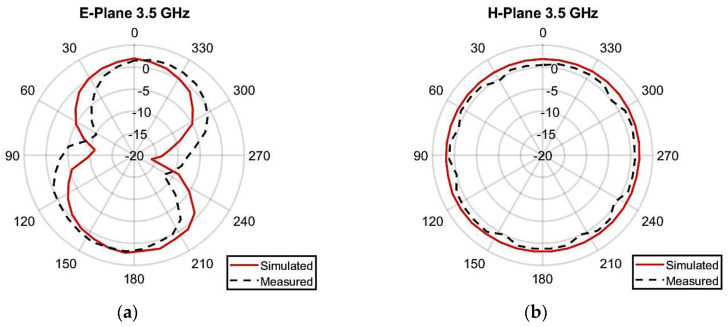

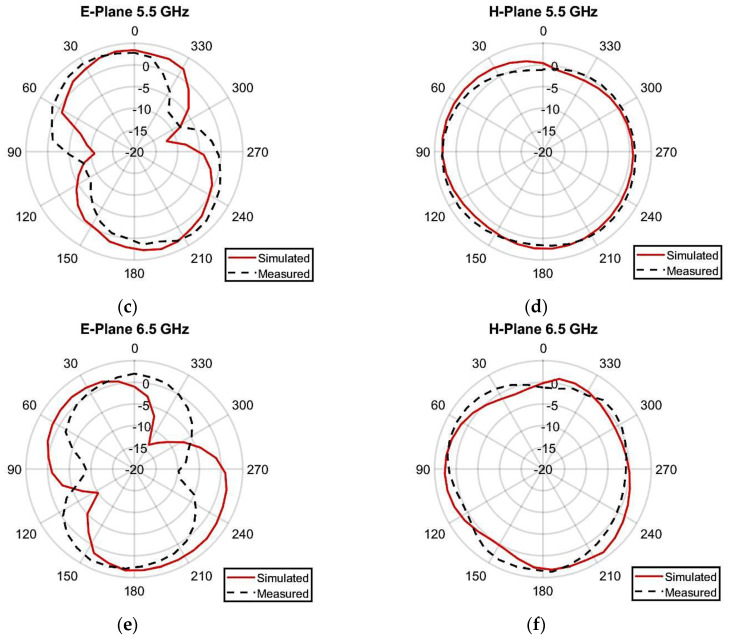
Radiation pattern of presented MIMO multiband antenna: (**a**) E-plane (3.50 GHz), (**b**) H-plane (3.50 GHz), (**c**) E-plane (5.50 GHz), (**d**) H-plane (5.50 GHz), (**e**) E-plane (6.50 GHz), (**f**) H-plane (6.50 GHz).

The radiation pattern of the antenna was measured by utilizing an anechoic chamber, as illustrated in Figure 11. The simulated and laboratory-measured radiation patterns of the presented two-element MIMO multiband antenna at 3.50 GHz, 5.50 GHz, and 6.50 GHz are presented in Figure 12. While testing, antenna 1 was connected to the setup, and a terminator was plugged into the port of antenna 2. At 3.5 GHz, the highest gain of 2.24 dBi was seen at 170 degrees, while the lowest gain of −13.8 dBi occurred at 260 degrees, as presented in Figure 12a. For 5.50 GHz, the peak gain value came out to be 3.49 dBi at 30 degrees, whereas the lowest gain of −12.2 dBi was seen at 280 degrees, as demonstrated in Figure 12c. At 6.50 GHz, a maximum gain of 3.41 dBi was observed at 170 degrees, while a minimum gain of −13.5 dBi occurred at 320 degrees, as shown in Figure 12e.

**Figure 13:**
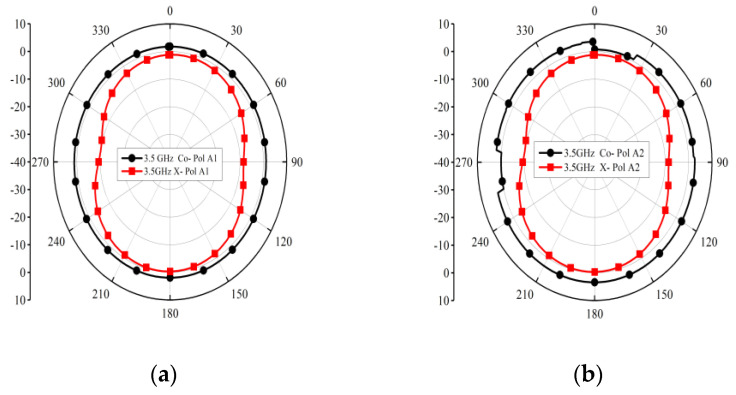

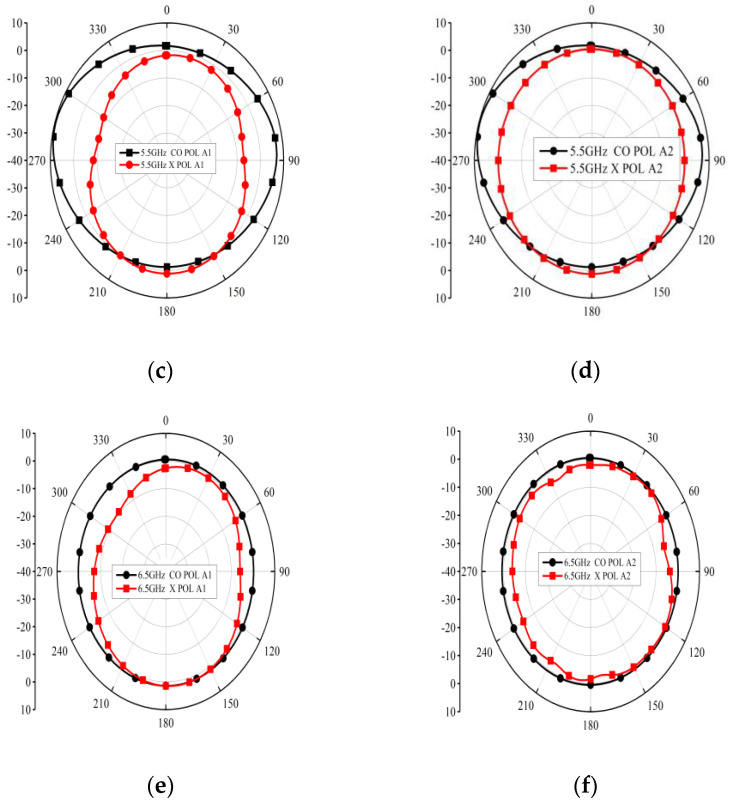
Co- and cross-polarization: (**a**) 3.5 GHz, Antenna 1; (**b**) 3.5 GHz, Antenna 2; (**c**) 5.5 GHz, Antenna 1; (**d**) 5.5 GHz, Antenna 2; (**e**) 6.5 GHz, Antenna 1; (**f**) 6.5 GHz, Antenna 2.

Figure 13 shows the simulated co- and cross-polarization results at 3.5 GHz, 5.5 GHz, and 6.5 GHz, respectively.

**Figure 14:**
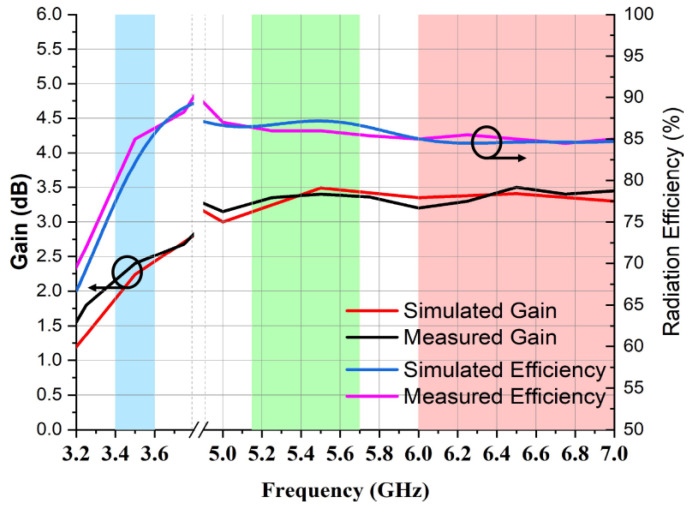
Radiation efficiency and gain of two-element MIMO multiband antenna.

Figure 14 illustrates the calculated radiation efficiency and gain at different frequencies of the MIMO antenna. The peak gain of around 3.49 dBi occurred at 5.5 GHz, while gain values of 2.24 dBi at 3.50 GHz and 3.41 dBi at 6.50 GHz were recorded. The observed radiation efficiency was higher than 80% throughout the operating band.

The diversity performance of the proposed two-element MIMO multiband antenna was assessed with the help of the parameters DG (diversity gain), ECC (envelope correlation coefficient), and TARC (total active reflection coefficient). It considers the radiation pattern, its polarization, and the antenna element’s relative phase. The ECC between two antenna elements in a MIMO is calculated with the help of the following mathematical model [19,20]:

(1) $$ECC=\frac{|∯E_{\theta i}.E_{\theta j}^{*}+E_{\emptyset i}\cdot E_{\emptyset j}^{*}dΩ{|}^{2}}{∯E_{\theta i}\cdot E_{\theta i}^{*}+E_{\emptyset i}.E_{\emptyset i}^{*}dΩ∯E_{\theta j}\cdot E_{\theta j}^{*}+E_{\emptyset j}\cdot E_{\emptyset j}^{*}dΩ}\;$$

where *i* and *j* indicate port numbers. *DG* is an important aspect that certifies the diversity performance of MIMO systems. It is calculated using the following equation [21,22]:

(2) $$DG=10\times \sqrt{1-\left|ECC\right|}\;$$

**Figure 15:**
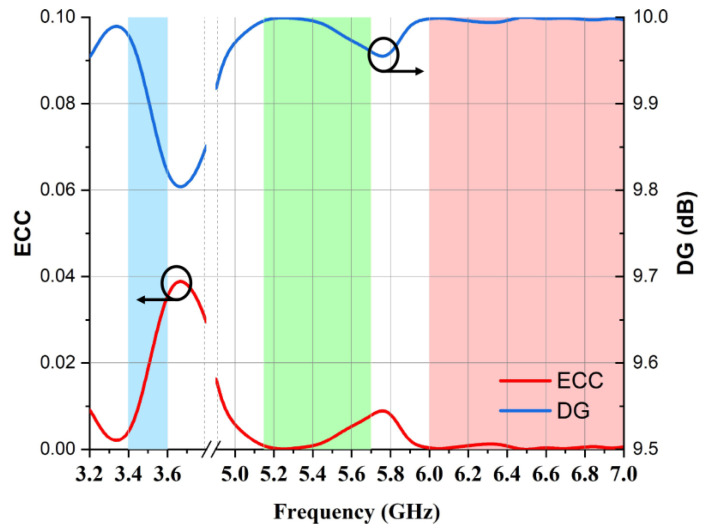
Calculated ECC and DG.

In Figure 15, it is notable that the value of the envelope correlation coefficient was below 0.04, while the diversity gain was well above 9.5 for the complete band of operations.

**Figure 16:**
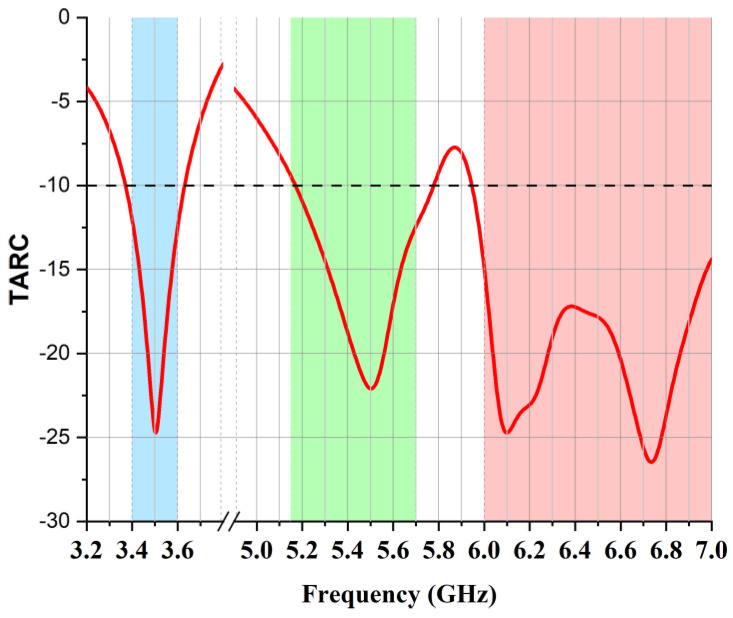
TARC of proposed two-element MIMO multiband antenna.

For calculating TARC, the laboratory-measured S-parameters of a two-element MIMO multiband antenna were used in the following mathematical relationship [23,24]:

(3) $$TARC=\frac{\sqrt{\sum_{i=1}^{2}|b_{i}{|}^{2}}}{\sum_{i=1}^{2}|a_{i}{|}^{2}}\;$$

where a_i_ denotes an incident wave and b_i_ denotes a reflected wave. It is evident in Figure 16 that TARC was below −10 dB in all the bands of operation.

The channel capacity loss (CCL) is another essential diversity parameter that defines the maximum attainable limit of the communication transmission rate. The CCL of the proposed MIMO antenna was measured using Equation (4) from [22]:

(4) $$C_{loss}=-log_{2}\det\left(\alpha^{R}\right)$$

$$Where,\;\alpha^{R}=\left[\begin{array}{ccc} \alpha_{11} & \cdots & \alpha_{14}\\ \vdots & \ddots & \vdots \\ \alpha_{41} & \cdots & \alpha_{44} \end{array}\right]$$

$$\mathrm{And}\;\alpha_{ii}=1-\left(\sum_{j=1}^{N}|S_{ij}{|}^{2}\right)$$

$$Also,\;\alpha_{ij}=-\left(S_{ij}*S_{ij}+S_{ji}*S_{ij}\right)$$

**Figure 17:**
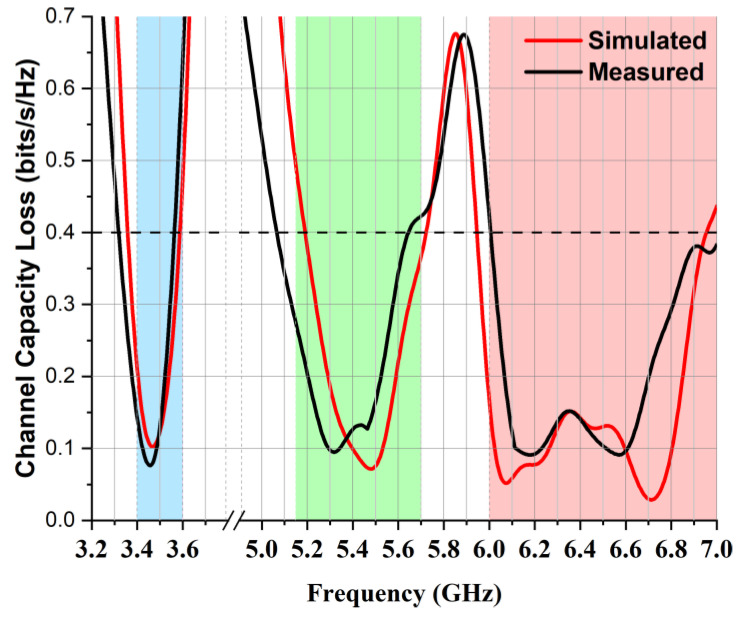
Channel capacity loss (CCL) of proposed two-element MIMO multiband antenna.

Its simulation results were compared with the measured results shown in Figure 17, in which it can be noted that the simulation and measured results of channel capacity loss were below 0.4 bits/s/Hz in the operating frequency bands, which satisfied the acceptable limit condition. A comparative analysis of the proposed MIMO antenna against the existing literature on MIMO antennas is presented in Table 2. The results indicate that the proposed MIMO antenna is competitive with the existing MIMO antennas. Notably, the proposed antenna demonstrated higher isolation than those in [4,9], lower values of ECC compared with those in [5,6,9], higher efficiency than those in [4,8,9], and higher gain than all those reported in the literature in the table [3,4,5,6,7,8,9]. Additionally, it has a reduced antenna size compared with those in [3,5,6,7,8,9]. The tabulated summary provides strong evidence that the presented research on MIMO multiband antennas is highly competitive, taking into account antenna gain, radiation efficiency, size, operating bands, diversity performance, and isolation.

## 5. Conclusions

This research effort presents a compact MIMO multiband antenna. The initial design architecture comprised a single element consisting of a 5G-shaped microstrip, two L-shaped strips, and a partial/semi-ground structure designed to radiate on the 3.50 GHz, 5.50 GHz, and 6.50 GHz frequency bands. For the MIMO, a duplicate design was placed in an orthogonal position with respect to the first element. The peak gain was recorded at 3.49 dB at 5.50 GHz, and the radiation efficiency was above 80% throughout the operating bands. ECC was observed below 0.04, DG was higher than 9.5 dB, and TARC was below −10 dB in all the bands of operation. In the presented research effort, the MIMO multiband antenna was miniaturized to a size of 16 × 28 × 1.6 mm^3^. The laboratory-measured findings are in good agreement with simulated results, indicating that the presented antenna is suitable for 5G, WLAN, and WiFi-6 applications.

## Figures and Tables

**Table 1:** Physical dimensions of presented MIMO multiband antenna.

Specification	Dimension (mm)	Specification	Dimension (mm)
L_1_	1	L_11_	1.5
W_1_	5	W_11_	7
L_2_	1.5	L_12_	2.8
W_2_	3.6	W_12_	2.5
L_3_	1.7	L_13_	1.8
W_3_	2.2	W_13_	0.8
L_4_	1.1	L_14_	8.8
L_5_	0.5	L_15_	0.5
W_5_	4.8	W_15_	6.5
L_6_	2.9	GL	2.5
W_6_	3.1	GW	10
L_7_	4.9	LS_1_	16
W_7_	1.3	WS_1_	10
L_8_	8.3	LS_2_	16
W_8_	0.6	WS_2_	28
L_9_	1.8	D_1_	3.3
W_9_	4.2	D_2_	2.8
L_10_	6.8	D_3_	12.70
W_10_	2		

**Table 2:** Summarized comparison of the presented research work with existing literature.

Ref.	Operating Frequency (GHz)	Isolation (dB)	ECC	Peak Gain (dBi)	Efficiency (%)	Decoupling Method	Total Size (mm^3^)	Implementation Complexity
[3]	2.25–2.41, 3.36–3.65 and 4.7–6.25	<−15	<0.0086	1.5–2.9	80–88	Self-isolated	48 × 48 × 1.6	Uncomplicated
[4]	3.35–3.67	<−10	NR	2	43–45.5	Ferrite loading	11.3 × 4 × 1	Complicated
[5]	4.36–6.90	<−20	<0.08	2	>90	Self-isolated	25 × 25 × 1.57	Uncomplicated
[6]	3.4–3.6 5.15–5.85	<−15	<0.11	2	>86	Parasitic stripline	25 × 25 × 1.57	Uncomplicated
[7]	3.1–5.2	<−15	<0.05	2.6	>90	Self-isolated	35 × 35 × 1.57	Uncomplicated
[8]	3.4–3.6	>15	<0.012	2.5	60–70	Parasitic structure	75 × 150 × 1.6 (8 elements)	Uncomplicated
[9]	3.4–3.8	>12	<0.05	3	65–80	Modified ground structure	75 × 150 × 1.59 (2 elements)	Uncomplicated
**This work**	**3.5, 5.5 and 6.5**	**>15**	**<0.04**	**3.49**	**>80**	**Polarization diversity**	**16 × 28 × 1.6 (2 elements)**	**Uncomplicated**

## Data Availability

All the data is available in this study.
